# Effect of Tytanit and Stymjod on *Phleum pratense* L. Photosynthetic Activity and the Content of Chlorophyll Pigments

**DOI:** 10.3390/plants14121814

**Published:** 2025-06-13

**Authors:** Jacek Sosnowski, Milena Truba, Elżbieta Malinowska, Paweł Kifer, Piotr Krasnodębski

**Affiliations:** Department of Agricultural Sciences, University of Siedlce, 14 Bolesława Prusa, 08-110 Siedlce, Polandmilena.truba@uws.edu.pl (M.T.); pk85318@stud.uws.edu.pl (P.K.); pk62@stud.uws.edu.pl (P.K.)

**Keywords:** growth biostimulant, growth regulator, chlorophyll a fluorescence

## Abstract

The use of Stymjod and Tytanit supports natural resistance mechanisms and improves the condition of Phleum pratense, which is in line with sustainable agriculture and integrated production. The aim of the research was to determine the effect of Stymjod and Tytanit and of the number of their sprays per growth cycle on chlorophyll fluorescence and the content of chlorophyll pigments in *Phleum pratense* L. leaf blades. The following research factors were used: Factor I—treatment: Control; Tytanit—0.04%; Stymjod—2.5%. Factor II—number of sprays: L1—plants sprayed one time; L2—plants sprayed two times. The use of the Tytanit regulator in the cultivation of *Phleum pratense* L. contributed to an increase in chlorophyll fluorescence parameters, i.e., qN and qP. In addition, an increase in chlorophyll a and b content was noted. The application of the Stymjod stimulator increased ΔF/Fm′. For the vast majority of chlorophyll a fluorescence parameters, higher values were noted when plants were sprayed two times per growth cycle, i.e., Fv/Fm, ΔF/Fm′, qN, chlorophyll pigments.

## 1. Introduction

Apart from plant protection products and fertilizers, a wide range of plant cultivation practices and innovations are introduced into sustainable crop production. A variety of growth stimulants such as Stymjod and Tytanit are used to control plant physiological processes affecting the growth and development of crops [1]. According to Wadas and Kalinowski [2], Tytanit is a liquid mineral growth biostimulant in the form of titanium ascorbate. Numerous literature reports describe a wide spectrum of its effects on ornamental plants [3], fruit crops [1], vegetables [4], and grasses and legume plants in permanent grassland [5,6,7,8,9]. According to Mackiewicz-Walec and Olszewska [10], Stymjod is a nanotechnology-based biostimulator that contains highly available mineral and organic nutrients that promote plant ontogeny. It is a growth stimulant, the positive effect of which has been confirmed by numerous scientific reports. So far it has been used in the cultivation of fruit crops [11], vegetables [12], and forage and lawn grasses [10,13], as well as ornamental plants [14].

Currently, there is a lack of studies in the existing research on Stymiod on the photosynthetic activity of *Phleum pratense*. The previous studies report a positive effect of Stymiod on the grass species *Dactylis glomerata*, where the plant treated with Stymiod, regardless of concentration, had a better efficiency of the photosynthetic apparatus. This was evidenced by the values of maximum photosystem efficiency (Fv/Fm), actual photosystem efficiency (ΔF/Fm′), and non-photochemical extinction coefficient (qN). All concentrations of Stymjod increased photosynthetic activity in plants [15]. Similar results were obtained in a study on the photosynthetic activity of Festuca glauca, which was due to an increase in photosynthetic pigments [14]. The positive effect of Stymjod on the development, biomass yield and physiological activity of plants indicates its usefulness in the cultivation of sorghum [15] and Jerusalem artichoke [16], which can reduce the use of synthetic fertilizers, benefit the environment, and reduce toxic substances in plants.

The existing research shows that the element Ti, which is the basic component of the Titanite formulation, stimulates the enzymatic activity of the plant and the photosynthetic process [17,18]. Titanite applied at high doses interacts with Fe in the electron transport chain, which causes a decrease in the efficiency of photosystem II [19]. According to Jajor et al. [20], plants treated with Titanite can be strengthened because the preparation stimulates physiological processes in them. As a result, plants are in better condition, are less susceptible to disease, and better able to withstand pest attacks.

Chlorophyll a fluorescence is an indicator of photosynthetic activity, which, in turn, is affected by other physiological processes and by the plant environment [21]. The leading parameter of the photosynthetic apparatus is the maximum photochemical efficiency (Fv/Fm) of photosystem II (PSII). In optimal growing conditions, the Fv/Fm of mature plants is about 0.85, but it may vary across plant species and varieties. Many authors agree that its significant decrease indicates plant stress [21,22,23]. According to the literature, the maximum photochemical efficiency of PSII can be used to determine the effect of stimulants on the physiological state of crops [24,25,26,27]. Among other chlorophyll fluorescence parameters, the following help to understand plant processes: the effective efficiency of PSII (ΔF/Fm′) and photochemical fluorescence quenching (qP) and non-photochemical fluorescence quenching (qN) coefficients. The ΔF/Fm′ value characterizes photosynthetic efficiency, related to the efficiency of electron transport [27,28]. The qP parameter is used to determine the proportion of light energy absorbed by PSII to the energy used for photosynthetic reactions by open centers [28]. In turn, the qN parameter is regulated by changes in the pH value on both sides of thylakoid membranes [29].

Chlorophyll a and b are plant pigments converting light energy into stored chemical energy [30]. According to the previous studies [31,32], chlorophyll content affects photosynthetic potential of the plant and allows the assessing of its nutritional status. In addition, chlorophyll content in the leaves provides valuable information about the physiological state and about the aging of the plant and its exposure to stress [33]. Assimilation pigments, such as chlorophylls, are among the most important chemical compounds in plants, as they affect the intensity of photosynthesis and biomass production [34]. Chlorophyll pigments determine the intensity of a plant’s color. Research by Radkowski et al. [35] showed that the formation of stronger shoots with longer stems and longer and wider leaves was due to increased chlorophyll content in the leaves. Plant nutrition affects chlorophyll content. Zielewicz et al. [34] deduced that higher soil richness in macronutrients such as Mg and P resulted in higher chlorophyll pigment content. A study by Radkowski and Radkowska [36] showed a beneficial effect of silicon additives on the agricultural traits of garden timothy of the Egida variety. During the study it was found that the relative content of chlorophyll increased during the growth period of the plant, and the yield of timothy seeds, germination capacity, and weight of 1000 seeds also increased. In addition, the work of Xie et al. [37] suggests that silicon-based fertilizers increase chlorophyll content, pure photosynthesis, and stomatal conductance in plant leaves.

Under stress conditions, the photosynthetic activity of the plant decreases. Instead of converting the maximum amount of energy to photosynthesis, the plant begins to deplete energy in the form of chlorophyll fluorescence. Reducing the physical size of the light-harvesting complex may also be another strategy to protect the photosystem [38]. The level of chlorophyll fluorescence affects plant productivity, which is limited by overly intense light, temperature changes, water deficiency, harmful effects of salts and pollutants in the soil, and elemental deficiency [38,39,40].

In existing studies, there is no information on the fluorescence process of chlorophyll a in photosystem II in the grass species Phleum pratense under the influence of the biostimulants Stymjod and Titanit. The aim of the research was to determine the effect of Stymjod and Tytanit and of the number of their sprays per growth cycle on chlorophyll fluorescence and the content of chlorophyll pigments in *Phleum pratense* L. leaf blades. The Fv/Fm, qN, and qP parameters were used to assess fluorescence, while chlorophyll pigments were evaluated on the basis of chlorophyll a and b concentration in plant leaf blades.

## 2. Results and Discussion

### 2.1. Photosynthetic Activity

**Figure 1:**
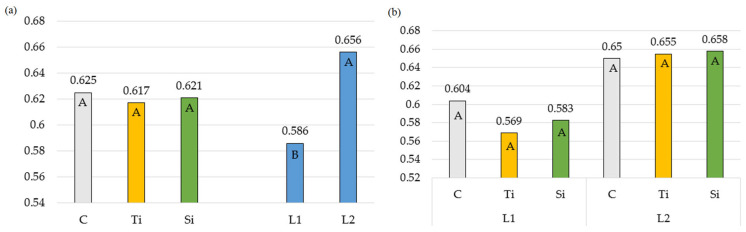
The maximum photosystem efficiency: (**a**) Averages for preparations and number of applications; (**b**) Double interaction between preparation and number of applications. The means in columns marked with the same uppercase letters do not differ significantly (*p* < 0.05). Abbreviations: C—control object; Ti—object with Tytanit; Si—object with Stymjod; L1—one application of preparation; L2—two applications of preparation.

Compared to control, the values of chlorophyll fluorescence induction parameters and chlorophyll content (Table 1, Table 2, Table 3 and Table 4) indicated a multidirectional effect of the applied products on the photosynthetic activity of *Phleum pratense* L. leaves. Fluorescence measurements were performed on plant leaves after darkroom relaxation. The maximum photosystem efficiency (Fv/Fm) was determined separately for each growth cycle (I—spring growth, II—summer growth, III—autumn growth). It varied statistically significantly only in response to the number of sprays (Figure 1a), but treatment did not affect its values. Throughout the growing seasons, the Fv/Fm ratio did not vary, either (Table 1, Figure 1b), and the demand of plants for products constituting the assimilatory power was steady, which did not cause disturbances in plant growth and development [41].

Increased values of the ratio meant activation of the photosystem in the darkroom adaptation state resulting from a lack of photoinhibition, otherwise occurring in nitrogen-deficient plant cells [42]. A rising Fv/Fm ratio means that the energy used to transport electrons is not reduced [43]. At the same time, according to Nishiyama et al. [44], an increase in the activity of reaction centers in the darkroom adaptation state is the result of supplying them with the right amount of nitrogen, which translates into high activity of the photosynthetic apparatus and increased efficiency of light energy conversion.

**Figure 2:**
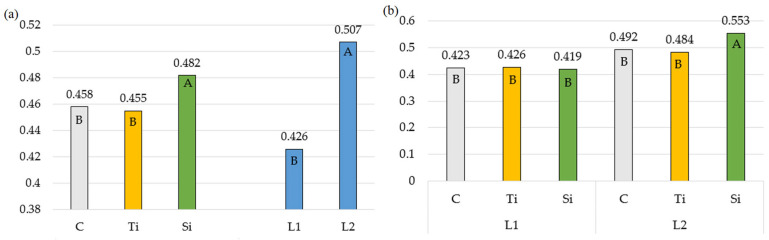
The effective efficiency of the photosystem: (**a**) Averages for preparations and number of applications; (**b**) Double interaction between preparation and number of applications. The means in columns marked with the same uppercase letters do not differ significantly (*p* < 0.05). Abbreviations: C—control object; Ti—object with Tytanit; Si—object with Stymjod; L1—one application of preparation; L2—two applications of preparation.

The effective efficiency of the photosystem (ΔF/Fm′) varied across growth cycles and was significantly affected by treatment and the number of sprays (Table 2, Figure 2b). The application of treatment two times each growth cycle resulted in an average 19% increase in the parameter value (Figure 2a).

The stimulants were the most effective when applied during the summer period (Growth II). The ΔF/Fm′ ratio was higher for *Phleum pratense* L. treated with Stymjod (0.482) than for plants treated with Tytanit. On the other hand, Sosnowski and Truba [45] recorded a 19% to 22% increase, depending on the dose, in the ΔF/Fm′ parameter of *Festulolium braunii* treated with Tytanit. Spraying with Stymjod twice increased the actual photochemical energy conversion quantum yield in photosystem II, which translates into the electron quantum yield of PSII. The ∆F/Fm′ reflects the photochemical quantum conversion function of a rather small layer of chloroplasts in the upper outer half of the leaf in that the parameter is sensitive to changes in the environment and responds rapidly with a decrease [46].

**Figure 3:**
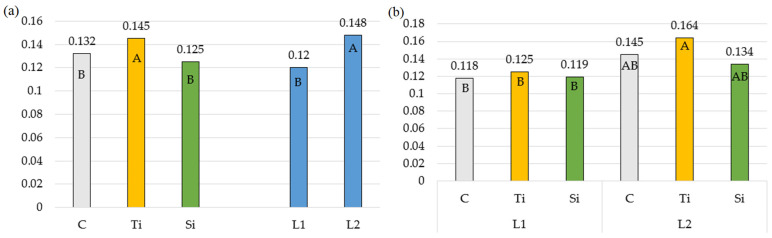
Non-photochemical fluorescence quenching coefficient: (**a**) Averages for preparations and number of applications; (**b**) Double interaction between preparation and number of applications. The means in columns marked with the same uppercase letters do not differ significantly (*p* < 0.05). Abbreviations: C—control object; Ti—object with Tytanit; Si—object with Stymjod; L1—one application of preparation; L2—two applications of preparation.

In the present experiment (Table 3, Figure 3), only the Tytanit regulator contributed to an increase in the non-photochemical fluorescence quenching coefficient (qN). Its value increased by an average of 13.3% in relation to units with Stymjod and to control. Similar results were reported by Sosnowski and Truba [45] in the cultivation of *Trifolium pratense*, with Tytanit increasing the coefficient (qN) by up to 25% depending on the regulator dose.

**Figure 4:**
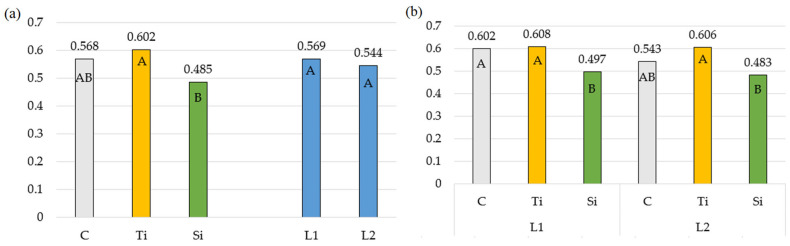
Photochemical fluorescence quenching: (**a**) Averages for preparations and number of applications; (**b**) Double interaction between preparation and number of applications. The means in columns marked with the same uppercase letters do not differ significantly (*p* < 0.05). Abbreviations: C—control object; Ti—object with Tytanit; Si—object with Stymjod; L1—one application of preparation; L2—two applications of preparation.

In like manner, the effect of Stymjod on the values of the photochemical fluorescence quenching (qP) was weaker than of Tytanit (Table 4, Figure 4). Similarly, Sosnowski et al. [12] investigated an effect of Stymjod used in the cultivation of *Dactylis glomerata* on this parameter and reported that none of the product doses significantly affected its value, ranging from 0.537 to 0.556.

The photochemical quenching factor qP is an index of the open centers of the PSII reaction. This parameter determines the ratio of light energy absorbed by PSII to the energy used by the open centers for photosynthetic reactions [28]. The ratio maintains higher values when photosynthetic activity is high. The value of the qP and qN coefficients has been shown to depend on the rate constant of all exciton-absorbing processes in PSII and, consequently, both parameters can be considered as indicators of photochemical and non-photochemical fluorescence quenching processes [47].

It is worth noting that the value of some parameters of plant photosynthetic activity depended on weather conditions. The average values of the non-photochemical fluorescence quenching coefficient (qN) were greater during the spring and autumn growth (with 0.137 and 0.138, respectively). As indicated by meteorological data (Table 7), the spring and autumn seasons of 2022 were characterized by periods with optimal (April 2022) and humid (September 2022) hydrothermal conditions. The exception was a very dry summer in 2022 affecting the qN value, with enough rainfall only in July. The effect of drought stress on chlorophyll fluorescence has also been discussed by other authors. Kiani et al. [48] argued that increasing water stress did not cause a long-term decrease in the photosynthetic parameters of *Helianthus annuus* L., but it reduced the actual electron transport efficiency of PSII. In addition, the QTL (the Quantitative Trait Loci) analysis conducted by the authors showed that several genomic regions take part in shaping chlorophyll fluorescence parameters during drought stress. In most cases, specific genetic loci were associated with a given stress condition. This suggested that the expression of photosynthesis-related genes varied under changing moisture conditions.

### 2.2. Chlorophyll Pigments

**Figure 5:**
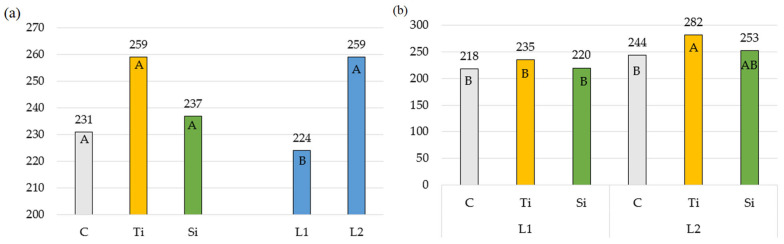
Chlorophyll a content: (**a**) Averages for preparations and number of applications; (**b**) Double interaction between preparation and number of applications. The means in columns marked with the same uppercase letters do not differ significantly (*p* < 0.05). Abbreviations: C—control object; Ti—object with Tytanit; Si—object with Stymjod; L1—one application of preparation; L2—two applications of preparation.

**Figure 6:**
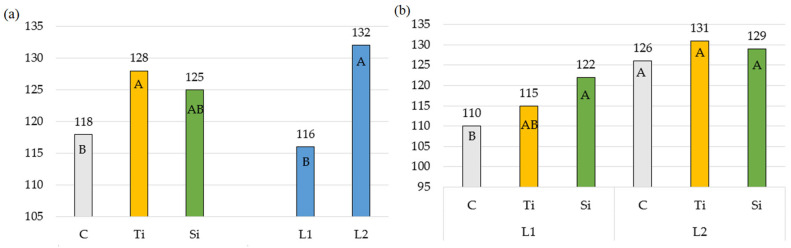
Chlorophyll b content: (**a**) Averages for preparations and number of applications; (**b**) Double interaction between preparation and number of applications. The means in columns marked with the same uppercase letters do not differ significantly (*p* < 0.05). Abbreviations: C—control object; Ti—object with Tytanit; Si—object with Stymjod; L1—one application of preparation; L2—two applications of preparation.

The content of chlorophyll a and b in *Phleum pratense* L. leaves (Table 5 and Table 6) significantly increased only in response to the Tytanit regulator. The increase was 10.7% for the content of chlorophyll a and 8.7% for the content of chlorophyll b (Figure 5a and Figure 6a). The concentration of chlorophyll pigments in the leaves was also affected by the number and time of spraying (Figure 5b and Figure 6b). Double application of the products resulted in an average 14.7% increase in pigment content, with the greatest value noted in summer. Photosynthetic pigments are responsible for collecting light and transferring it to the photosynthetic reaction centers, so their concentration is related to photosynthesis efficiency. Thus, an increase in the content of pigments may contribute to an increase in photosynthetic activity [49,50]. Therefore, as noted in the present research, the Tytanit regulator increased the concentration of chlorophyll pigments of *Phleum pratense* L. leaves and, at the same time, increased their photosynthetic activity (Table 5 and Table 6).

Similar results were recorded by Wadas and Kalinowski [2], who investigated the effect of Tytanit on the assimilation area of leaves and chlorophyll content in very early potato cultivars (*Solanum tuberosum*). The stimulating effect of titanium ions delivered to the leaves in the form of the Tytanit growth regulator on the leaf assimilation area and chlorophyll content in potato was observed. After applying Tytanit, the plants produced a larger leaf assimilation area, especially during stressful conditions. In addition, according to studies conducted in China [51], after triple application of foliar fertilizer containing titanium, potato leaves were dark green, shiny, and dense, which was also confirmed by another experiment with Tytanit applied to *Medicago* × *varia* T. Martyn. Other authors [52,53] observed that the application of Tytanit stimulated chlorophyll content in the leaves of timothy (*Phleum pratense* L.), winter wheat (*Triticum aestivum* L.), and winter rape (*Brassica napus* L.). In addition, the authors reported that the Tytanit dose and date of application slightly affected chlorophyll content in the leaves.

Kováčik et al. [53] demonstrated a positive effect of double spraying with Mg-Tytanit on chlorophyll content in winter wheat and winter rape leaves. It was higher in plants treated with a Mg-Tytanit dose of 0.2 dm^3^ ha^−1^ than in plants treated with 0.4 m^3^ ha^−1^. However, the third spraying of plants with Mg-Tytanit doses tended to reduce the chlorophyll content in the leaves. On the other hand, the present research also indicated that the content of chlorophyll pigments in the leaves of *Phleum pratense* L. depended on weather conditions. The lowest concentration of chlorophyll a and b was noted in spring. As shown by the distribution of Sielianinov’s coefficient (Table 7), the spring and autumn seasons in 2023 were characterized by severe rainfall shortages. At that time, extremely dry (late July and September) and very dry (April) months were recorded. Drought stress during that period significantly decreased pigment content in plant leaves. The lowest chlorophyll a content (233 mg 100 g^−1^ fresh weight) was recorded in spring, 11.2% lower than in summer. Similarly, the concentration of chlorophyll b in the leaves was most strongly reduced by spring droughts, with a decrease of 14.6% in relation to summer. The effect of drought stress on the content of chlorophyll a and b in crops has also been reported by other authors. Kiani et al. [48] observed that chlorophyll a and b content in sunflower (*Helianthus annuus* L.) leaves decreased with increasing soil moisture deficit. Reductions in chlorophyll content in cotton (*Gossypium hirsutum* L.) leaves under drought conditions were also reported by Massacci et al. [54]. Similar results were reported by Arji et al. [55] in their experiment on the effect of drought stress on selected physiological parameters of Olea europaea. However, the average chlorophyll content was statistically significantly greater, compared to control, in years with extremely dry periods.

**Table 7:** Air temperature, precipitation, and Sielianinov’s coefficient throughout 2022 and 2023 growing periods.

Year	Month						
	Apr	May	June	July	Aug	Sept	Oct
Average daily air temperature (°C)							
2022	7.0	13.6	19.9	19.3	21.0	11.7	10.6
2023	8.7	13.4	18.0	20.3	21.3	18.0	10.4
Means	7.4	13.1	19.4	20.8	19.8	14.2	9.9
1996–2010	8.0	13.5	17.0	19.7	18.5	13.5	7.9
Monthly precipitation (mm)							
2022	31.5	31.1	26.5	95.7	39.3	64.9	13.9
2023	12.4	46.5	53.6	31.4	25.0	16.6	32.8
Means	28.6	35.7	38.0	59.0	53.2	41.2	17.5
1996–2010	33.6	58.3	59.6	57.5	59.9	42.3	24.2
Sielianinov’s coefficient (K)							
2022	1.50 (o)	0.74 (d)	0.44 (vd)	1.60 (o)	0.60 (vd)	1.85 (fh)	0.42 (vd)
2023	0.48 (vd)	1.12 (fd)	0.99 (d)	0.50 (vd)	0.38 (ed)	0.31 (ed)	1.02 (fd)

Abbreviation: K-value—period: ≤0.40—extremely dry (ed), 0.41–0.70—very dry (vd), 0.71–1.00—dry (d), 1.01–1.30—fairly dry (fd), 1.31–1.60—optimal (o), 1.61–2.00—fairly humid (fh), 2.01–2.50—humid (h), 2.51–3.0—very humid (vh), >3.00—extremely humid (eh). Source: own elaboration.

According to Hussain et al. [56], foliar application of ionic Ti increased root morphological parameters, which may be useful in increasing nutrient and water uptake. The increased biomass accumulation in Ti-treated plants was mainly due to an increase in chlorophyll pigments (a, b, and a + b). However, caution should be exercised in the application of Ti. According to Hruby et al. [57], Ti can replace iron (Fe) and magnesium (Mg) at their binding sites. The effect of Ti on Fe uptake induces Fe deficiency, and the consequence is biochemical and activity changes in the photosynthetic apparatus, i.e., lower content of light-harvesting chlorophyll pigments and disconnection of the antenna in photosystem II (PSII) [20]. Stymjod contains nitrogen, phosphorus, potassium, and numerous micronutrients as its main constituents [13]. Nitrogen is the main component of chlorophyll and cell membranes, among others. Nitrogen deficiency causes a decrease in transpiration, stomatal conductance and the electron acceptor pool in PSII. In turn, various phosphorus compounds are involved in photosynthesis and respiration reactions, providing the osmotic potential of the cell sap and playing an important role in cellular energy metabolism. Potassium plays a key role in cellular osmoregulation. Potassium is required to maintain the pH gradient between the inner and outer sides of the thylakoid membrane [58,59].

**Figure 7:**
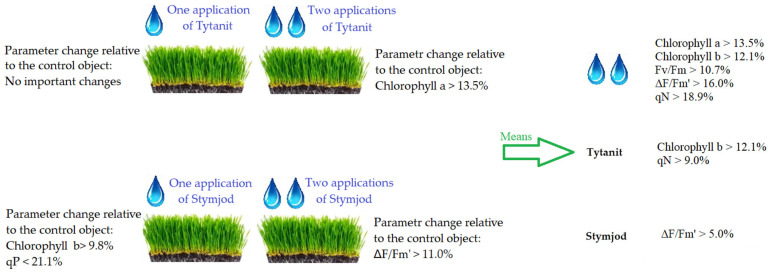
Summary of the most relevant results for comparison between treatments. Abbreviations: qP—photochemical fluorescence quenching coefficient; qN—non-photochemical fluorescence quenching coefficient; ΔF/Fm’—effective efficiency of PSII; Fv/Fm—maximum photosystem efficiency; “>”—value increased relative to the control object; “<”—value decreased relative to the control object; Mean for 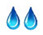
—percentage difference in value between one and two biostimulator applications; Mean for Tytanit—percentage difference in value between the control object and the objects with the Titanit application; Mean for Stymjod—percentage difference in value between the control object and the objects with the Stymjod application.

To summarize the main results, a comparison of the effects of the different treatments on the plant is shown in Figure 7.

## 3. Materials and Methods

### 3.1. Plant Growth Conditions and Experimental Design

The research was conducted on the basis of a ring experiment at the Prof. Feliks Ceglarek Agricultural Experimental Station of the University of Siedlce (52°10′03″ N; 22°17′24″ E) between 2022 and 2023. In April 2022 polyurethane rings with a diameter of 20 cm and a height of 20 cm were dug to a depth of 16 cm with a spacing of 0.8 × 0.8 m and filled with native soil. The space between the rings was covered with a mat to stop weeds from growing. Then 10 grass seeds were sown by hand into each ring. During the third–fourth leaf stage, the seedlings were assessed and the weakest ones were removed. Thus, in each ring three *Phleum pratense* L. plants cv. Prosna were left. According to the producer (DANKO Hodowla Roślin Ltd., Chorynia, Poland), this variety is intended for mowing. Its characteristic feature is a very high production potential. It can be grown throughout the country, on moist soils rich in nutrients. In addition, it is characterized by good winter hardiness and increased resistance during summer droughts. It is recommended for permanent grassland mixtures and for field cultivation.

The grass was harvested three times a year during the 2022–2023 growing periods. The experiment was conducted in four replications, and its design was as follows:

Factor I—treatment:

- Control—plants sprayed with distilled water;
- Tytanit—plants sprayed with 0.04% Tytanit solution;
- Stymjod—plants sprayed with 2.5% Stymjod solution.
- Factor II—number of sprays:
- L1—plants sprayed one time per growth cycle at the stage of stem formation;
- L2—plants sprayed two times per growth cycle at the stage of stem formation and 10 days afterwards.

According to the manufacturer, Titanite (Intermag, Olokusz, Poland) contains 8.5 g Ti 1 dm^−3^ (0.8% *m*/*m*) in the form of Ti-ascorbate. The composition of Stymiod (Jeznach, Sochaczew, Poland), according to the manufacturer, is as follows: macronutrients (N-6.3%; P-4.58%; K-6.42%; Mg-1.69%; S-1.6%), micronutrients (B-0.46%; Cu-0.17%; Fe-0.14%; Mn-0.16%; Mo-0.028%; Zn-0.42%), humic acids and amino acids. Both preparations are classified as biostimulants under European law [60]. The products (Tytanit and Stymjod) were applied during each growth cycle, in spring, summer, and autumn. Per each ring, 50 mL of liquid was used, spraying the plants until they were thoroughly wet.

The characteristics determined in plant material were as follows:

- Maximum efficiency of the photosystem (Fv/Fm);
- Effective efficiency of the photosystem (ΔF/Fm′);
- Non-photochemical fluorescence quenching coefficient (qN);
- Photochemical fluorescence quenching coefficient (qP);
- Chlorophyll a content in plant leaf blades (mg 100 g^−1^ fresh weight);
- Chlorophyll b content in plant leaf blades (mg 100 g^−1^ fresh weight).

### 3.2. Chlorophyll Pigments and Photosynthetic Activity

Leaf blades from the 3rd–4th node of randomly selected plant shoots were used to determine pigment content. Three samples of plant material were collected from each experimental unit. Chlorophyll a and b content was measured by the method of Arnon et al. [61], modified by Lichtenthaler and Wellburn [62]. The optical density of supernatants was determined by the Marcel Mini spectrophotometer with wavelengths of 440, 465, and 663 nm. The concentration of chlorophyll a and b was calculated according to the following formulas:

Chlorophyll a = [12.7 (E 663) − 2.69 (E 645)] w/v(1)

Chlorophyll b = [22.9 (E 645) − 4.68 (E 663)] w/v(2)

where

E is the quenching at a specific wavelength;

v is the amount of 80% acetone (cm^3^) used for extraction;

w is the weight of the sample (g).

The photosynthetic activity of plants was assessed by measuring chlorophyll fluorescence induction with the PAM 2000 Portable Fluorometer (Heinz Walz GmbH, Effeltrich, Germany). All measurements were performed on *Phleum pratense* L. well-developed leaves with five replicates. A 2030-B leaf-clip and a light-emitting diode with a wavelength of 650 nm and a standard intensity of 0.15 μmol m^−2^ s^−1^ PAR were used.

### 3.3. Meteorological Conditions

Meteorological data of the University of Siedlce Meteorological Station located at the Prof. Feliks Ceglarek Agricultural Experimental Station in Zawady confirmed dynamic weather patterns resulting from climate change (Table 7). In order to determine the temporal variability of meteorological conditions and their impact on plant growth and development, Sielianinov’s hydrothermal coefficient (K) was calculated on the basis of monthly precipitation (P) and the monthly sum of daily air temperatures (t), using the following formula [63]:

K = P/0.1Σt(3)

The values of Selianinov’s coefficient for each month of the growing period are presented in Table 7. Throughout the experiment, optimal hydrothermal conditions for the growth and development of plants were recorded only in April and July 2022, with quite favorable weather in May and October 2023. During both growing periods, moisture shortages prevailed, with very dry and dry spells. The scanty rainfall also resulted in soil moisture deficit in August and September 2023. The weather was fairly humid only in September 2022.

### 3.4. Soil Conditions

The experiment was established on rusty soil developed on sandy sediments [64]. Before sowing the seeds, soil samples were collected and dried at room temperature. After separating soil skeleton from fine earth on a sieve with a mesh of 2 mm holes, the following were determined:

- Granulometric composition (grain size)—by Casagrande’s hydrometric method modified by Prószyński [65];
- pH in a solution of 1 mol KCl dm^−3^ (pH_KCl_)—by the potentiometric method [66];
- Total carbon content—by the elemental analysis method, using the PerkinElmer 2400 Series II CHNS/O Elemental Analyzer, on the basis of which the content of soil organic matter was calculated, using the conversion factor of 1.724 [67];
- Content of N-NO_3_ and N-NH_4_—by the Kjeldahl method, after extraction in 1 mol KCl [68];
- Phosphorus, potassium, calcium, and magnesium content (available forms)—by the ICP-OES (Inductively Coupled Plasma Optical Emission Spectroscopy) method, using the Perkin-Elmer Optima 8300 emission spectrometer, after the extraction of components from the soil, using the Mehlich 3 method [69].

According to laboratory tests, the soil was slightly acidic (pH_KCl_ = 5.8), with organic matter content of 2.08% and the following average content of available forms of nitrogen and minerals (mg kg^−1^): 30.3 N-NO_3_; 49.0 N-NH_4_; 81.6 P; 182.4 K; 417.2 Ca; 66.1 Mg.

### 3.5. Statistical Analysis

The results were processed statistically using the analysis of variance ANOVA for a multivariate (years) experiment in a split-plot system with repeated measures (across harvests), in four replications and with a control series. The significance of the effect of experimental factors on the characteristics was determined using the Fisher–Snedecor F test. The significance of the differences between means was verified by Tukey’s post hoc test, also known as the HSD (Honestly Significant Difference) test. The significance level was set at *p* < 0.05. The Statistica 13–2017 program was used for the calculations. In the tables, letters were used to mark homogeneous groups. Means marked with the same letters in rows/columns do not differ significantly.

## 4. Conclusions

The use of the Tytanit regulator in the cultivation of *Phleum pratense* L. contributed to an increase in chlorophyll fluorescence parameters, i.e., the non-photochemical fluorescence quenching coefficient (qN) and the photochemical fluorescence quenching coefficient (qP). In addition, an increase in chlorophyll a and b content was noted. The application of the Stymjod stimulator to *Phleum pratense* L. increased the effective efficiency of the photosystem (ΔF/Fm’). For the vast majority of chlorophyll a fluorescence parameters, higher values were noted when plants were sprayed two times per growth cycle, i.e., maximum photosystem efficiency (Fv/Fm), effective photosystem efficiency (ΔF/Fm’), and the non-photochemical fluorescence quenching coefficient (qN), as well as the content of chlorophyll pigments. The values of the characteristics also changed depending on the growth cycle and hydrothermal conditions. Higher values of chlorophyll a and b were noted for the second harvest, during dry and very dry conditions. On the other hand, chlorophyll a fluorescence parameters, i.e., Fv/Fm, qN, and qP, were higher in plants of the first and third harvests, with optimal or wet conditions during the growing period. Research into the efficacy and mechanism of action of Titanite and Stymjod is expanding our knowledge of plant physiology and opening up new possibilities for integrated management of forage grass crops. Due to the origin of the ingredients and mechanism of action, Stymjod and Titanite are seen as more environmentally friendly solutions than classical chemicals. This makes them safe for the end consumer.

## Figures and Tables

**Table 1:** Effect of Tytanit and Stymjod treatment and the number of sprays per growth cycle on the maximum photosystem efficiency of *Phleum pratense* L. leaf blades.

Number of Sprays	Treatment	Growth Cycle/Harvest		
		I	II	III
L1	Control	0.621 Aa	0.570 Aa	0.622 Aa
	Tytanit	0.565 Ba	0.571 Aa	0.571 Ba
	Stymjod	0.567 Ba	0.565 Aa	0.618 ABa
L2	Control	0.643 Aa	0.641 Aa	0.656 Aa
	Tytanit	0.661 Aab	0.648 Ab	0.689 Aa
	Stymjod	0.679 Aa	0.641 Ab	0.653 Aa
Means for the number of sprays				
L1		0.584 Ba	0.569 Ba	0.604 Ba
L2		0.660 Aa	0.643 Aa	0.665 Aa
Means for treatment				
Control		0.632 Aa	0.605 Aa	0.639 Aa
Tytanit		0.613 Aa	0.609 Aa	0.630 Aa
Stymjod		0.623 Aa	0.603 Aa	0.636 Aa
Means		0.623 a	0.606 a	0.635 a

The means in columns marked with the same uppercase letters do not differ significantly (*p* < 0.05). The means in the rows marked with the same lowercase letters do not differ significantly (*p* < 0.05).

**Table 2:** Effect of Tytanit and Stymjod treatment and the number of sprays per growth cycle on the effective photosystem efficiency of *Phleum pratense* L. leaf blades.

Number of Sprays	Treatment	Growth Cycle/Harvest		
		I	II	III
L1	Control	0.403 Cb	0.452 Ba	0.413 Bb
	Tytanit	0.403 Cb	0.454 Ba	0.420 Bb
	Stymjod	0.414 Bab	0.440 Ba	0.403 Bb
L2	Control	0.429 Bb	0.594 Aa	0.457 Ab
	Tytanit	0.457 Bb	0.563 Aa	0.432 Ab
	Stymjod	0.593 Aa	0.559 Aa	0.480 Ab
Means for the number of sprays				
L1		0.407 Ba	0.459 Ba	0.412 Ba
L2		0.493 Aab	0.572 Aa	0.456 Ab
Means for treatment				
Control		0.416 Bb	0.523 Aa	0.435 Ab
Tytanit		0.430 Ab	0.509 Aa	0.426 Ab
Stymjod		0.504 Aa	0.500 Aa	0.442 Ab
Means		0.450 b	0.511 a	0.434 b

The means in columns marked with the same uppercase letters do not differ significantly (*p* < 0.05). The means in the rows marked with the same lowercase letters do not differ significantly (*p* < 0.05).

**Table 3:** Effect of Tytanit and Stymjod treatment and the number of sprays per growth cycle on the non-photochemical fluorescence quenching coefficient (qN) of *Phleum pratense* L. leaf blades.

Number of Sprays	Treatment	Growth Cycle/Harvest		
		I	II	III
L1	Control	0.118 Ca	0.116 Ba	0.119 Ba
	Tytanit	0.125 Ca	0.122 Ba	0.128 Ba
	Stymjod	0.121 Ca	0.115 Ba	0.116 Ba
L2	Control	0.149 Ba	0.135 Ab	0.152 ABa
	Tytanit	0.176 Aa	0.145 Ab	0.170 Aa
	Stymjod	0.130 Ba	0.133 Aa	0.140 ABa
Means for the number of sprays				
L1		0.121 Ba	0.118 Ba	0.121 Ba
L2		0.152 Aa	0.138 Ab	0.154 Aa
Means for treatment				
Control		0.134 ABa	0.126 Ab	0.136 ABa
Tytanit		0.151 Aa	0.134 Ab	0.149 Aa
Stymjod		0.126 Ba	0.124 Aa	0.128 Ba
Means		0.137 a	0.128 b	0.138 a

The means in columns marked with the same uppercase letters do not differ significantly (*p* < 0.05). The means in the rows marked with the same lowercase letters do not differ significantly (*p* < 0.05).

**Table 4:** Effect of Tytanit and Stymjod treatment and the number of sprays per growth cycle on the photochemical fluorescence quenching coefficient (qP) of *Phleum pratense* L. leaf blades.

Number of Sprays	Treatment	Growth Cycle/Harvest		
		I	II	III
L1	Control	0.623 Aa	0.566 Ab	0.615 Aa
	Tytanit	0.606 Aa	0.576 Ab	0.642 Aa
	Stymjod	0.466 Ba	0.523 Aa	0.502 Ba
L2	Control	0.530 ABa	0.565 ABa	0.533 ABa
	Tytanit	0.615 Aa	0.566 Ab	0.638 Aa
	Stymjod	0.466 Ba	0.513 Ba	0.471 Ba
Means for the number of sprays				
L1		0.565 Aa	0.555 Aa	0.587 Aa
L2		0.537 Aa	0.548 Aa	0.547 Aa
Means for treatment				
Control		0.577 Aa	0.561 Aa	0.575 Ba
Tytanit		0.611 Aa	0.566 Ab	0.640 Aa
Stymjod		0.466 Ba	0.513 Aa	0.487 Ca
Means		0.551 a	0.552 a	0.567 a

The means in columns marked with the same uppercase letters do not differ significantly (*p* < 0.05). The means in the rows marked with the same lowercase letters do not differ significantly (*p* < 0.05).

**Table 5:** Effect of Tytanit and Stymjod treatment and the number of sprays per growth cycle on chlorophyll a content (mg 100 g^−1^ fresh weight) of *Phleum pratense* L. leaf blades.

Number of Sprays	Treatment	Growth Cycle/Harvest		
		I	II	III
L1	Control	225 BCa	224 Ba	206 Ca
	Tytanit	252 Ba	273 Aa	179 Cb
	Stymjod	184 cc	263 Aa	213 Bb
L2	Control	249 Ba	246 ABa	236 Ba
	Tytanit	285 Aa	274 Aab	289 Ab
	Stymjod	203 Cb	272 Aa	283 Aa
Means for the number of sprays				
L1		220 Bb	253 Aa	199 Bc
L2		246 Ab	264 Aa	269 Ab
Means for treatment				
Control		237 Ba	235 Ba	221 Bb
Tytanit		269 Aa	274 Aa	234 Cb
Stymjod		194 cc	268 Aa	248 Ab
Means		233 b	259 a	234 b

The means in columns marked with the same uppercase letters do not differ significantly (*p* < 0.05). The means in the rows marked with the same lowercase letters do not differ significantly (*p* < 0.05).

**Table 6:** Effect of Tytanit and Stymjod treatment and the number of sprays per growth cycle on chlorophyll b content (mg 100 g^−1^ fresh weight) of *Phleum pratense* L. leaf blades.

Number of Sprays	Treatment	Growth Cycle/Harvest		
		I	II	III
L1	Control	108 CBa	117 Cb	106 Ca
	Tytanit	119 Bb	126 Ba	101 Cb
	Stymjod	100 Cb	144 Aa	131 Aa
L2	Control	117 Bb	131 ABa	129 Ba
	Tytanit	140 Aa	147 Aa	135 Aa
	Stymjod	110 Cb	139 ABa	138 Aa
Means for the number of sprays				
L1		109 Bb	126 Ba	113 Bb
L2		122 Ab	139 Aa	134 Aa
Means for treatment				
Control		113 Bb	124 Ba	118 Bb
Tytanit		130 Aa	137 Aa	118 Bb
Stymjod		105 Cb	137 Aa	135 Aa
Means		116 b	133 a	124 ab

The means in columns marked with the same uppercase letters do not differ significantly (*p* < 0.05). The means in the rows marked with the same lowercase letters do not differ significantly (*p* < 0.05).

## Data Availability

The datasets used and/or analyzed during the current study are available from the corresponding author on reasonable request.
